# Driving Time, Distance, and Cost to Access Syringe Services Programs in the US

**DOI:** 10.1001/jamanetworkopen.2026.9753

**Published:** 2026-04-29

**Authors:** Spruha Joshi, Mengni Jing, Katherine Wheeler-Martin, Pooja Shah, Corey S. Davis, Charles J. DiMaggio, Magdalena Cerdá

**Affiliations:** 1 Department of Epidemiology, School of Public Health, University of Michigan, Ann Arbor; 2 Division of Epidemiology, Department of Population Health, NYU Grossman School of Medicine, New York, New York; 3 Network for Public Health Law, Edina, Minnesota; 4 Department of Surgery, NYU Grossman School of Medicine, New York, New York

## Abstract

**Importance:**

Syringe services programs (SSPs) are evidence-based interventions that reduce bloodborne infections and injection-related harms among people who inject drugs, yet access remains limited and geographically uneven across the US.

**Objective:**

To quantify the travel time, distance, and cost required to reach the nearest SSP from population-weighted census tracts nationwide and to examine differences by urbanicity, state, and SSP legality.

**Design, Setting, and Participants:**

This cross-sectional geospatial study linked all known SSP locations as of August 2024 to the population-weighted centroids of census tracts in the 50 US states and the District of Columbia. Analyses were conducted between December 2024 and February 2026.

**Main Outcomes and Measures:**

Population-weighted mean and median driving time, distance, and cost to access the nearest SSP, stratified by National Center for Health Statistics urban-rural county category and SSP legal status. Costs were estimated using 2024 Internal Revenue Service (IRS) medical mileage deduction rates and 2022 state-specific gasoline prices.

**Results:**

In 1338 SSPs across 83 780 census tracts, the population-weighted mean 1-way driving time to the nearest SSP was 46.1 minutes (95% CI, 45.7-46.5 minutes) and the median was 23.3 minutes (IQR, 12.2-58.5 minutes). Altogether, 23.1% of the population lived more than 60 minutes from an SSP and 12.6% lived over 120 minutes away. The mean 1-way driving distance was 41.8 miles (95% CI, 41.3-42.2 miles). The mean 1-way driving cost was $8.77 (95% CI, $8.68-$8.86) using the 2024 IRS mileage rate and $6.91 (95% CI, $6.84-$6.98) using state mean gasoline prices in 2022. In states where SSPs were legal, mean driving time was 30.1 minutes (95% CI, 29.8-30.4 minutes) and mean cost by IRS mileage rates was $4.94 (IQR, $4.88-$5.00), compared with 110.7 minutes (95% CI, 109.6-111.8 minutes) and $24.19 (IQR, $23.92-$24.46) in states where SSPs were illegal.

**Conclusions and Relevance:**

This cross-sectional study of travel burden to SSPs found substantial geographic and financial barriers to accessing SSPs across the US, particularly in nonmetropolitan areas. Targeting new SSPs to areas with the greatest travel burden could improve utilization and reduce drug-related morbidity.

## Key Points

**Question:**

What travel time, distance, and cost burdens are associated with accessing the nearest syringe services program (SSP) in the US, and do these vary by SSP legality?

**Findings:**

In this cross-sectional study including 1338 SSPs, the population-weighted mean 1-way driving time to the nearest SSP was 46 minutes and cost was $9, with 23% of the population living more than 60 minutes away. In states legalizing SSPs, mean 1-way travel time and cost were 30 minutes and $5 vs 111 minutes and $24 in states where they were illegal.

**Meaning:**

The findings suggest limited geographic access to SSPs imposes time and financial burdens that may reduce utilization and exacerbate drug-related harms.

## Teaser

This cross-sectional study examines travel time, distance, and cost to reach the nearest syringe services program (SSP) from centroids of population-weighted census tracts across the US and whether there are differences by urbanicity, state, and SSP legality.

## Introduction

The US continues to grapple with the overdose crisis, with more than 80 000 drug overdose deaths reported in 2024.^1^ Drug-related morbidities also continue to rise. In 2023, there were 69 000 estimated acute hepatitis C virus (HCV) infections, largely driven by syringe sharing.^2^ Meanwhile, skin and soft tissue infections also affect a large share of persons who inject drugs (lifetime prevalence, 27%-69%).^3^ This burden highlights the importance of evidence-based solutions, including syringe services programs (SSPs) that address both the immediate and long-term consequences of drug use.^4^ SSPs are public health programs that provide access to sterile injection equipment, smoking supplies, fentanyl test strips, referrals to substance use treatment, and naloxone.^4^ They also are important tools for reducing injection-related harms and improving engagement in care.^4,5,6^

Despite the proven benefits of SSPs, access remains uneven and inaccessible in many parts of the country.^7,8,9,10^ The impact of SSPs is contingent on their availability and accessibility. Barriers such as long travel distances and inadequate transportation infrastructure can severely limit access.^11^ Unequal access to SSPs may exacerbate geographic disparities in use of SSPs,^12^ stigma may be more pronounced, and there also may be a general lack of access to health care.^11,13^

Most research on geographic access to SSPs has focused on physical proximity or time traveled, often within a single state or limited set of cities. Studies have shown considerable variation in travel burden. For example, mean driving time to the nearest harm reduction facility was 16 minutes in Alabama,^14^ while driving distance to the closest SSP ranged from 0.69 to 6.03 miles across 17 US metropolitan areas.^15^ A national study of young persons living with HCV found that the median distance from zip code centroids to the nearest SSP was 37 miles.^16^ These studies focused on single states or only quantified travel burden in terms of time and distance, missing the important measure of cost.

Quantifying the cost of accessing an SSP both in monetary terms and time expenditure can offer a more comprehensive understanding of the structural and individual burdens imposed by limited SSP accessibility. To address these gaps, our study quantified driving times, distances, and costs from population-weighted centroids of all census tracts in the US to the nearest SSP to better estimate the overall burden in accessing SSPs and capture how driving distances vary by overdose rates. We compared differences across urbanicity, states, and SSP legality.

## Methods

### Data Sources

In this cross-sectional study, addresses of SSPs were obtained from the North American Syringe Exchange Network (NASEN)^17^ and state health department websites as of August 2024. We included all listed addresses and intersections regularly served by fixed and mobile programs and city or county centroids for mobile programs regularly serving unlisted locations within cities and counties. If a single program listed multiple locations served, we counted all unique reported addresses. We included post office boxes if they were the only address available for a program but excluded them if physical addresses were listed for the same program. We deduplicated addresses found in more than 1 source or served by more than 1 program. This study followed the Strengthening the Reporting of Observational Studies in Epidemiology (STROBE) reporting guideline for cross-sectional studies. It was deemed exempt from approval and informed consent by the University of Michigan institutional review board because the study did not involve human participants.

County categories were classified as metropolitan or nonmetropolitan according to the 2023 National Center for Health Statistics (NCHS) Urban Rural Classification Scheme.^18^ Large central metropolitan counties were those encompassing the entire population of a principal city, entirely contained in the largest principal city, or with at least 250 000 residents of any principal city within a metropolitan statistical area (MSA) of at least 1 million residents. Large fringe metropolitan counties were other metropolitan counties within MSAs of at least 1 million residents that did not meet large central metropolitan criteria. Medium metropolitan counties were in MSAs with populations of 250 000 to 999 999, and small metropolitan counties were in MSAs with populations of 50 000 to 249 999. Nonmetropolitan counties were designated as micropolitan if located within a micropolitan statistical area and noncore otherwise. Counties were further classified into census regions and divisions following the 2020 US Census framework: Northeast (New England and Middle Atlantic divisions), Midwest (East North Central and West North Central divisions), South (South Atlantic, East South Central, and West South Central divisions), and West (Mountain and Pacific divisions).^19^

Population-weighted centroids of census tracts were obtained from the 2020 US Census Bureau geographies.^20^ The centers of population at the census tract level were calculated based on the 2020 census population and 2020 census tract shapefiles.

Census tracts were classified based on whether their state permitted (“legal”) or prohibited (“illegal”) the distribution of drug use supplies from SSPs.^21^ Notably, although Idaho was classified as illegal in July 2025 data, it was considered legal during our study period.

We estimated the smoothed county-level relative risk of drug overdose deaths from 2019 to 2023 using a Besag-York-Mollié spatial model implemented in the INLA package of R, version 4.4.1 (R Project for Statistical Computing),^22,23,24,25^ using detailed multiple-cause-of-death microdata files from NCHS and aggregating counts of overdose deaths by county of occurrence. Drug overdose deaths were defined as records having an *International Statistical Classification of Diseases and Related Health Problems, Tenth Revision (ICD-10)* underlying cause of death code of X40 to X44, X60 to X64, X85, or Y10 to Y14.^26^ Relative risks were modeled with population offsets using American Community Survey 5-year county total population estimates from 2019 to 2023.^27^ We then categorized counties into tertiles based on the posterior mean of the smoothed relative risks, moving from lower than expected to higher than expected relative risk of overdose.

### Geocoding

We conducted batch geocoding for the SSP address list using ArcGIS Pro software, version 3.3.2 (esri), with the StreetMap Premium North American Q3 2025 locators.^28^ All SSP addresses were geocoded to either the PointAddress or the StreetAddress, which include either house numbers and street names or street centerlines. Incomplete addresses were excluded (eFigure 1 in Supplement 1). Of census tracts in the 50 states and the District of Columbia with corresponding population-weighted centroids, we excluded population-weighted centroids that were not within 3 miles of the street network, thus constraining our ability to estimate driving time and distance. We also excluded census tracts with no population reported and those for which we were unable to measure a driving route to any SSP. In addition, we excluded tracts in Alaska that had driving routes longer than 12 hours to the closest SSP from the main analysis.

### Driving Time and Distance

We used the origin destination cost matrix^29^ with the routing service^30^ in StreetMap Premium to calculate the travel time and travel distance from each census tract population-weighted centroid to the closest SSP. Travel time was computed based on the mean of observed vehicle speeds over the past year,^30^ with a searching tolerance of 3 miles.

### Driving Cost

Driving cost of each driving route from the census tract centroid to the closest SSP address was calculated by multiplying the travel distance estimates from the driving analysis with 2 types of cost rates. First, each state’s cost rate per 1 mile for light-duty vehicles was computed based on the mean fuel efficiency of 22.8 miles per gallon in the US in 2022^31^ and the mean cost of motor gasoline per state in 2022.^32^ Second, the deductible cost of operating an automobile for medical purposes was 21 cents per mile in 2024, according to the standard mileage rates issued by the Internal Revenue Service (IRS).^33^

### Statistical Analysis

The population-weighted driving time, driving distance, and driving cost at the county level and at the state level were calculated by weighting each census tract population by the total population among all census tracts that were included in the final analysis within each county category and within each state. The distribution of unweighted driving time varied across NCHS county categories and did not satisfy the normality assumption required for parametric tests. Therefore, we used the Kruskal-Wallis test, a nonparametric alternative to analysis of variance, to examine whether unweighted driving time and unweighted driving cost differed across NCHS county categories. All statistical tests were 2-tailed, with significance set at *P* < .05. Access measures were stratified by state SSP legal status, classifying tracts as located in states where SSPs were legal vs illegal. All analyses were conducted at the census tract level. Bivariate maps used the 2024 US Census county boundary shapefiles (n = 3104 counties). Data were unavailable for counties affected by boundary changes where the NCHS maintains older geographic definitions and for counties with small populations (<500 residents) or incomplete mortality coverage in NCHS data for 2019 to 2023. Counties were grouped into 9 groups by tertiles of the median driving time to the closest SSP in 2024 and tertiles of the relative risk of drug overdose deaths from 2019 to 2023. Analyses were conducted between December 2024 and February 2026. Maps were created and descriptive statistics were calculated using R, version 4.4.2.

## Results

A total of 1495 SSP addresses were geocoded, from which 157 incomplete addresses were excluded, leaving 1338 SSP addresses in the final analysis. Of 84 414 census tracts in the 50 states and the District of Columbia with corresponding population-weighted centroids, we excluded 214 not within 3 miles of the street network; we also excluded 352 census tracts with no population reported and 48 for which we were unable to measure a driving route to an SSP, and we excluded 20 in Alaska with driving routes of more than 12 hours to the nearest SSP (representing 0.02% of the US population). The final analytic sample size of census tracts was 83 780 (eFigure 2 in Supplement 1).

### Driving Time

**Figure 1.**
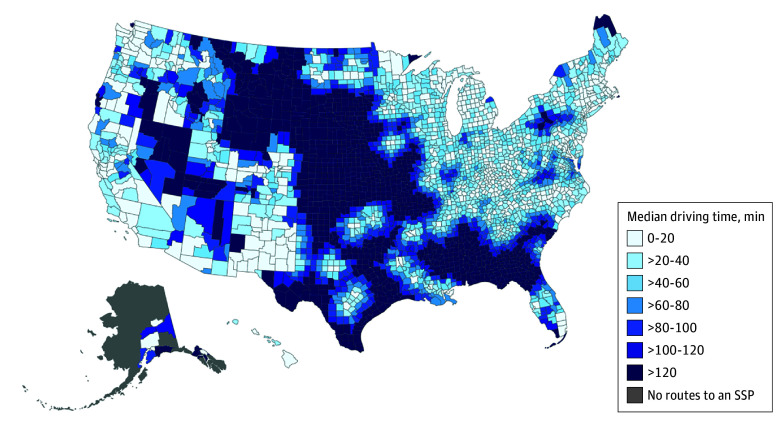
Median Population-Weighted 1-Way Driving Times to the Closest Syringe Services Program (SSP) at the County Level, 2024

The population-weighted mean 1-way driving time to the nearest SSP was 46.1 minutes (95% CI, 45.7-46.5 minutes), and the median 1-way driving time was 23.3 minutes (IQR, 12.2-58.5 minutes) (Table 1). Overall, 40.2% of the US population lived more than 30 minutes from an SSP, 23.1% more than 60 minutes, 17.1% more than 90 minutes, and 12.6% more than 120 minutes (Figure 1). Five states—Kansas, Mississippi, Nebraska, South Dakota, and Wyoming—had no SSPs. Population-weighted median driving times exceeded 120 minutes in 7 states (Alabama, Kansas, Missouri, Nebraska, South Dakota, Texas, and Wyoming) (eTable 1 in Supplement 1). Similar patterns were observed for mean driving times. Driving times varied substantially across NCHS urban-rural categories (Kruskal-Wallis χ^2^_5_, 13 704; *P* < .001). Mean 1-way driving time was 31.8 minutes (95% CI, 31.1-32.4 minutes) in large central metropolitan counties vs 87.6 minutes (95% CI, 85.9-89.3 minutes) in noncore counties (Table 1). Correspondingly, 18.2% of residents in large central metropolitan counties lived more than 30 minutes from an SSP and 12.3% more than 60 minutes away, compared with 84.0% and 56.2%, respectively, in noncore counties.

**Table 1.** One-Way Mean and Median Driving Times and Distances to the Closest SSP^a^

Census tracts (N = 83 780)	1-Way driving time, min		1-Way driving distance, miles	
	Population-weighted mean (95% CI)	Median (IQR)	Population-weighted mean (95% CI)	Median (IQR)
Total US	46.1 (45.7-46.5)	23.3 (12.2-58.5)	41.8 (41.3-42.2)	15.4 (5.5-50.5)
NCHS urban-rural county classification				
Metropolitan (n = 70 008)				
Large central (n = 25 529)	31.8 (31.1-32.4)	14.0 (8.2-22.2)	27.5 (26.8-28.3)	6.5 (2.5-14.2)
Large fringe (n = 19 256)	38.0 (37.4-38.7)	24.6 (16.0-38.7)	31.7 (31.0-32.5)	16.1 (8.1-29.5)
Medium (n = 17 666)	51.7 (50.9-52.6)	26.8 (12.6-79.4)	48.3 (47.3-49.3)	18.6 (6.4-75.1)
Small (n = 7557)	60.2 (58.7-61.7)	41.3 (14.1-89.2)	57.5 (55.8-59.1)	33.8 (7.9-86.2)
Nonmetropolitan (n = 13 772)				
Micropolitan (n = 7900)	68.6 (67.2-70.1)	51.4 (25.1-96.3)	64.8 (63.2-66.3)	44.0 (17.8-91.0)
Noncore (n = 5872)	87.6 (85.9-89.3)	71.6 (41.0-127.6)	84.1 (82.2-86.0)	63.6 (32.2-125.2)
SSP legality				
Legal (n = 66 845)	30.1 (29.8-30.4)	19.1 (10.7-36.0)	23.5 (23.3-23.8)	11.4 (4.4-27.3)
Illegal (n = 16 935)	110.7 (109.6-111.8)	122.8 (49.8-161.5)	115.2 (113.9-116.5)	127.8 (40.3-175.4)

Abbreviations: NCHS, National Center for Health Statistics; SSP, syringe services program.

^a^ Population-weighted mean (95% CI) and median (IQR) driving times and distances were calculated from census tract mean centers of population to the SSP with the shortest driving time.

### Driving Distance

The population-weighted mean 1-way driving distance to the nearest SSP was 41.8 miles (95% CI, 41.3-42.2 miles), and the median 1-way driving distance was 15.4 miles (IQR, 5.5-50.5 miles) (Table 1). The maximum 1-way driving distance was 385.3 miles. More than 60% of the US population lived more than 10 miles from an SSP, including 32.6% living more than 30 miles away and 23.7% more than 50 miles away. Like driving times, driving distances differed significantly across NCHS urban-rural categories (Kruskal-Wallis χ^2^_5_, 14 901; *P* < .001). Mean driving distance was 27.5 miles (95% CI, 26.8-28.3 miles) in large central metropolitan counties compared with 84.1 miles (95% CI, 82.2-86.0 miles) in noncore counties (Table 1).

### Driving Cost

**Figure 2.**
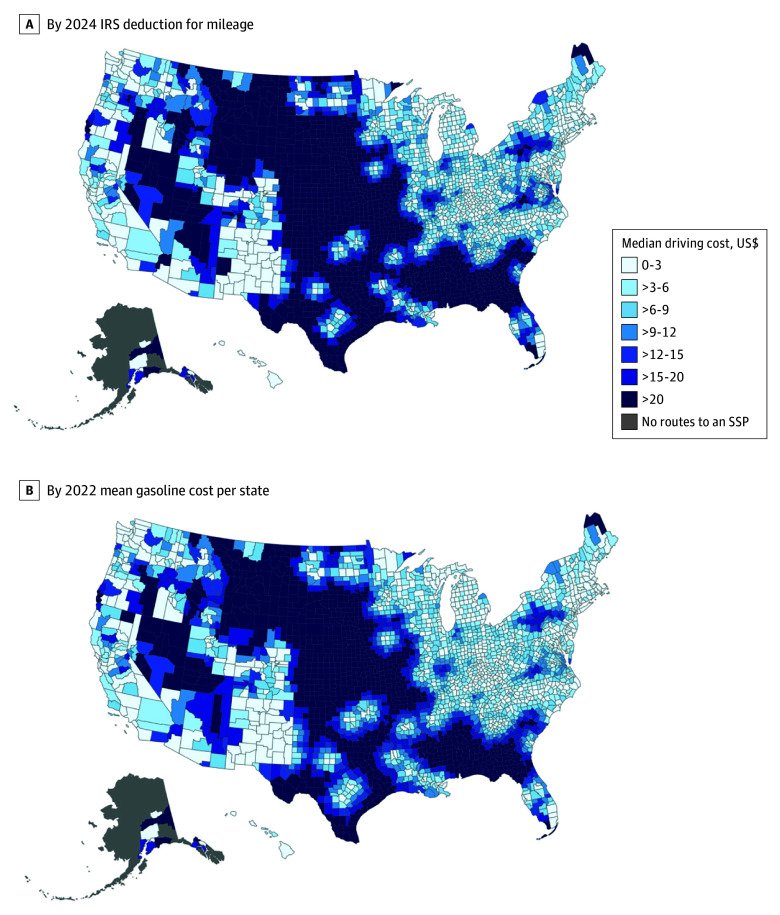
Median Population-Weighted 1-Way Driving Costs to the Closest Syringe Services Program (SSP) at the County Level, 2024 IRS indicates Internal Revenue Service.

The population-weighted mean 1-way driving cost to the nearest SSP was $8.77 (95% CI, $8.68-$8.86) using 2024 IRS medical mileage rates and $6.91 (95% CI, $6.84-$6.98) using 2022 state mean gasoline prices (Table 2). Median costs were $3.22 (IQR, $1.15-$10.61) and $2.76 (IQR, $0.99-$8.68), respectively. Maximum 1-way costs were $80.90 (IRS mileage) and $68.64 (gasoline cost). Using IRS mileage rates, 37.7% of the US population had a driving cost of more than $5.00 to an SSP and 24.5% had a cost of more than $10.00; corresponding estimates using gasoline prices were 33.6% and 21.6%, respectively (Figure 2). Costs differed significantly across NCHS urban-rural categories for both IRS mileage estimates (Kruskal-Wallis χ^2^_5_, 14 901; *P* < .001) and gasoline cost estimates (Kruskal-Wallis χ^2^_5_, 14 183; *P* < .001). Mean IRS-based cost was $5.78 (95% CI, $5.63-$5.93) in large central metropolitan counties compared with $17.66 (95% CI, $17.26-$18.07) in noncore counties (Table 2).

**Table 2.** One-Way Mean and Median Driving Costs and Fuel Costs to the Closest SSP^a^

Census tracts (N = 83 780)	1-Way cost estimate, US $			
	Driving, using 2024 IRS deduction for mileage		Fuel, using 2022 state mean cost of gasoline	
	Population-weighted mean (95% CI)	Median (IQR)	Population-weighted mean (95% CI)	Median (IQR)
Total US	8.77 (8.68-8.86)	3.22 (1.15-10.61)	6.91 (6.84-6.98)	2.76 (0.99-8.68)
NCHS urban-rural county classification				
Metropolitan (n = 70 008)				
Large central (n = 25 529)	5.78 (5.63-5.93)	1.37 (0.53-2.99)	4.55 (4.44-4.66)	1.22 (0.47-2.72)
Large fringe (n = 19 256)	6.67 (6.52-6.82)	3.38 (1.70-6.20)	5.25 (5.13-5.36)	2.80 (1.41-4.99)
Medium (n = 17 666)	10.15 (9.94-10.35)	3.91 (1.34-15.77)	7.93 (7.78-8.09)	3.37 (1.14-12.47)
Small (n = 7557)	12.07 (11.72-12.41)	7.11 (1.66-18.11)	9.60 (9.33-9.88)	5.94 (1.39-14.15)
Nonmetropolitan (n = 13 772)				
Micropolitan (n = 7900)	13.60 (13.28-13.93)	9.24 (3.74-19.11)	10.81 (10.56-11.07)	7.47 (3.10-15.31)
Noncore (n = 5872)	17.66 (17.26-18.07)	13.36 (6.77-26.29)	13.96 (13.64-14.28)	10.55 (5.55-20.65)
SSP legality				
Legal (n = 66 845)	4.94 (4.88-5.00)	2.40 (0.92-5.74)	3.80 (3.75-3.84)	2.08 (0.80-4.81)
Illegal (n = 16 935)	24.19 (23.92-24.46)	26.84 (8.46-36.82)	17.82 (17.63-18.02)	20.27 (6.97-27.56)

Abbreviations: NCHS, National Center for Health Statistics; SSP, syringe services program.

^a^ Population-weighted mean (95% CI) and median (IQR) driving times and distances were calculated from census tract mean centers of population to the SSP with the shortest driving time.

In a sensitivity analysis including the 20 census tracts in Alaska with driving times longer than 12 hours, results were similar to the main analysis. Any differences in state-specific driving times and costs were in Alaska only (eTables 2-4 in Supplement 1).

### SSP Legal Status

Access to SSPs differed markedly by legal status. In states where SSPs were legal (66 845 census tracts [79.8%]), the population-weighted mean 1-way driving time was 30.1 minutes (95% CI, 29.8-30.4 minutes) and mean distance was 23.5 miles (95% CI, 23.3-23.8 miles). In contrast, in states where SSPs were illegal (16 935 census tracts [20.2%]), mean driving time was 110.7 minutes (95% CI, 109.6-111.8 minutes) and mean distance was 115.2 miles (95% CI, 113.9-116.5 miles). Financial burdens followed similar patterns. Using 2024 IRS mileage rates, mean 1-way cost was $4.94 (95% CI, $4.88-$5.00) in states where SSPs were legal compared with $24.19 (95% CI, $23.92-$24.46) where they were illegal; corresponding estimates using state gasoline prices were $3.80 (95% CI, $3.75-$3.84) and $17.82 (95% CI, $17.63-$18.02), respectively (Table 2). Median values for time, distance, and cost showed comparable disparities, indicating substantially greater geographic and financial barriers to SSP access in states where SSPs were illegal.

### Rate of Overdose and SSP Driving Time

**Figure 3.**
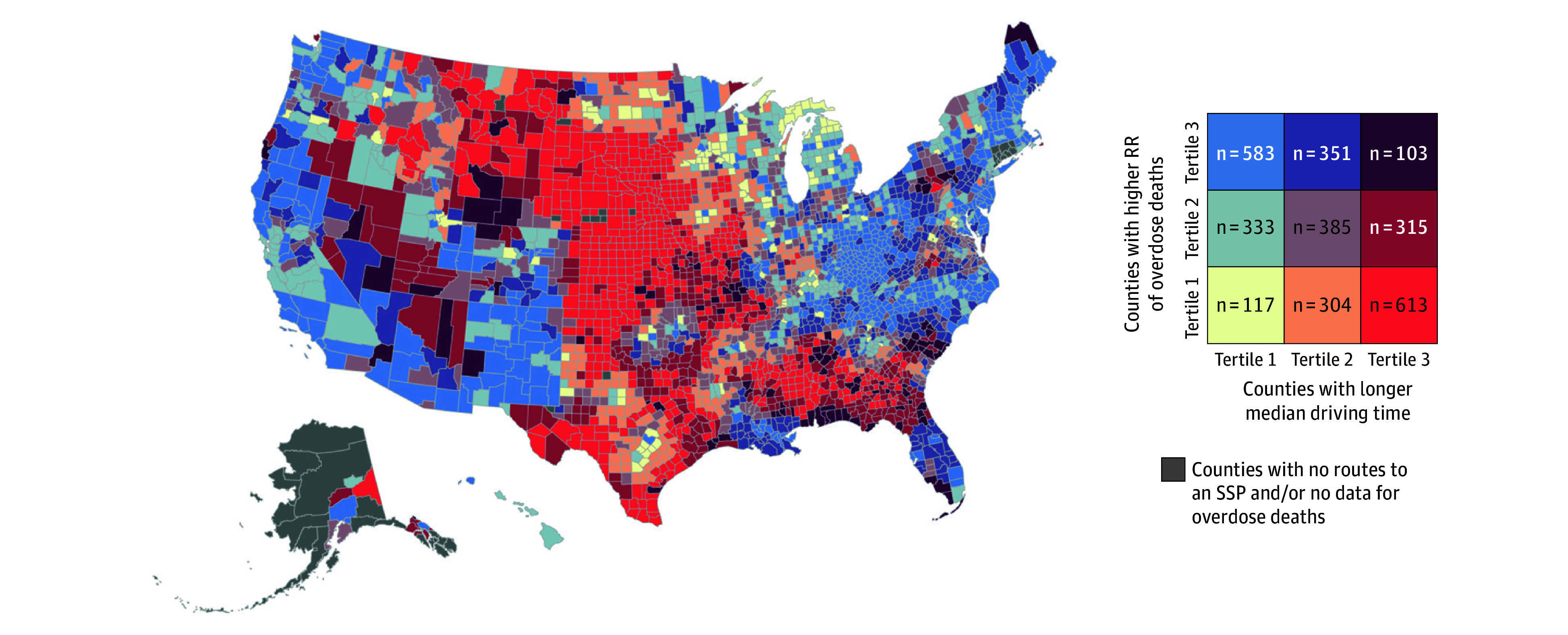
Bivariate Map for Median 1-Way Driving Time to the Closest Syringe Services Program (SSP) and Relative Risk (RR) of Overdose Deaths at the County Level Counties were grouped by tertiles of lowest (tertile 1) to highest median driving time to the closest SSP in 2024 and of lowest (tertile 1) to highest RR of drug overdose deaths from 2019 to 2023.

Among the 1037 counties in the highest tertile of overdose rates, 103 (9.9%) had median driving times ranging from 99.6 to 608.5 minutes and another 351 (33.8%) had median drive times ranging from 38.8 to 99.6 minutes. The 117 counties with the shortest median driving times (range of 3.0 to 38.8 minutes) also had the lowest overdose risk (Figure 3). These patterns highlight geographic inequities, with many rural and central US regions facing both elevated overdose risk and limited proximity to SSPs.

## Discussion

Across the US, access to SSPs was geographically uneven, with substantial portions of the population facing long travel times and nontrivial costs to reach the nearest SSP. By quantifying access nationally and examining time, distance, and cost simultaneously, this study extends prior work that has largely relied on localized or single-state analyses.^14,15,16^ A substantial proportion of the US population faced considerable geographic barriers to SSP access: 40.2% lived more than 30 minutes, 23.1% lived more than an hour, 17.1% lived more than 90 minutes, and 12.6% lived more than 2 hours from the nearest program. Our findings also highlight transportation cost as an underexamined but meaningful barrier to SSP access. Even modest 1-way travel costs accumulate for individuals requiring frequent syringe access, naloxone refills, or drug checking services. For people who use drugs, many of whom experience housing instability, unemployment, or poverty, travel costs of $15 to $25 round trip may represent a significant structural barrier. Cost-related SSP access burdens may therefore function similarly to other forms of financial stress in health care, constraining service utilization even when programs are legally permitted.

We found not only substantial heterogeneity across regions and urbanicity but also pronounced disparities by state legalization status, with residents of states where SSPs were illegal facing several-fold greater travel times and costs. People living in states with no SSPs (Kansas, Mississippi, Nebraska, South Dakota, and Wyoming) are required to cross state lines to access the nearest SSP. In states where SSPs were illegal, residents faced markedly longer travel times and distances and significantly higher out-of-pocket travel costs compared with states where SSPs were legal. These findings suggest that legal prohibitions may create meaningful obstacles to harm reduction access, particularly in rural and nonmetropolitan areas where baseline access challenges are already pronounced. These findings are particularly concerning given the urgent need to scale up harm reduction services in response to the overdose crisis. As fentanyl and other synthetic opioids continue to dominate the illicit drug supply, access to drug checking equipment, naloxone, and linkage to care becomes even more critical. SSPs are uniquely positioned to provide these services, yet their effectiveness is contingent on being geographically accessible to the populations who need them most. Limited geographic access to SSPs has significant and measurable public health consequences for people who inject drugs (PWID).

Prior work has consistently shown that distance from an SSP is a critical determinant of unsafe syringe-sharing practices,^15,34^ higher rates of syringe reuse,^35^ less probability of SSP utilization,^36^ and less access to syringes from SSP sources.^37^ Specifically, risk behaviors including syringe reuse and equipment sharing rise sharply as distance increases. Individuals living within a 30-minute drive of an SSP had a 47% higher rate of syringe reuse compared with those living within walking distance, and those living farther than a 30-minute drive had a 55% higher rate compared with those living within walking distance.^35^ Similarly, among PWID, each additional mile separating one’s residence from an SSP corresponded to a 6% higher likelihood of obtaining syringes from non-SSP sources.^37^ The effects of distance extend beyond syringe access to broader health outcomes. PWID living farther than 3 to 10 miles from fixed-site SSPs also experienced elevated HCV seroprevalence (25% higher) compared with those living within 1 mile.^34^ These studies suggest inadequate SSP access perpetuates unsafe injection practices, infectious disease transmission, and a lack of engagement with other harm reduction services. This is in line with other research suggesting that greater travel times and distances to care are associated with worse health outcomes; longer travel times and higher transportation costs can delay care seeking, reduce treatment adherence, and exacerbate existing inequities.^11,38^ To put our findings into context, 15.9% of US residents were found to lack 60-minute trauma center access,^39^ compared with 23.1% in this study who lived more than 60 minutes from an SSP. This suggests that geographic barriers to SSP access may exceed those observed for trauma center access nationally. Likewise, the drive times reported herein are longer compared with average travel to preventive health services even when considering rural areas, in which the mean drive time was found to be 18 minutes.^40^

### Limitations

Limitations of this study include potential underreporting or outdated information in publicly available SSP directories and our exclusion of SSPs that only provided mail-based services or outreach without fixed addresses. Second, while driving time, distance, and cost are critical measures, they do not capture other barriers such as stigma, legal restrictions, or hours of operation, which may further limit access to SSPs. Driving routes in this study were estimated from census tract population weighted centroids rather than individual residences of people who use drugs. Modeled travel times may underestimate actual travel, particularly for individuals relying on public transportation or traveling during peak traffic hours.

## Conclusions

This cross-sectional study highlights substantial geographic inequities in SSP access, with long travel times and high costs likely reducing utilization and increasing preventable harm. Improving equitable coverage will require more than expanding legal authorization of fixed-site programs; strategic deployment of mobile SSP units, pharmacy-based syringe access, secondary distribution, and mail-based services may be necessary to reduce structural access barriers.^41^

## Notes

Supplement 1. **eFigure 1.** Flowchart of SSP Addresses Included in the Final Analysis **eFigure 2.** Flowchart of Population-Weighted Centroids of Census Tracts Included in the Final Analysis **eTable 1.** Population-Weighted Driving Times and Distances to the Closest SSPs at the State Level Excluding Census Tracts With Routes That Take Longer Than 12 Hours **eTable 2.** Population-Weighted Driving Costs to the Closest SSPs at the State Level Excluding Census Tracts With Routes That Take Longer Than 12 Hours **eTable 3.** Driving Times and Driving Distances to the Closest SSPs Without Excluding Census Tracts With Routes That Take Longer Than 12 Hours **eTable 4.** Driving Costs to the Closest SSPs Without Excluding Census Tracts With Routes That Take Longer Than 12 Hours

Supplement 2.
Data Sharing Statement
