# Supplementary material for: Driving Time, Distance, and Cost to Access Syringe Services Programs in the US
Source: JAMA Netw Open. 2026 Apr 29;9(4):e269753. doi: 10.1001/jamanetworkopen.2026.9753 (PMC13129881; doi:10.1001/jamanetworkopen.2026.9753)
Supplement: Supplement 1. — eFigure 1. Flowchart of SSP Addresses Included in the Final Analysis eFigure 2. Flowchart of Population-Weighted Centroids of Census Tracts Included in the Final Analysis eTable 1. Population-Weighted Driving Times and Distances to the Closest SSPs at the State Level Excluding Census Tracts With Routes That Take Longer Than 12 Hours eTable 2. Population-Weighted Driving Costs to the Closest SSPs at the State Level Excluding Census Tracts With Routes That Take Longer Than 12 Hours eTable 3. Driving Times and Driving Distances to the Closest SSPs Without Excluding Census Tracts With Routes That Take Longer Than 12 Hours eTable 4. Driving Costs to the Closest SSPs Without Excluding Census Tracts With Routes That Take Longer Than 12 Hours [file jamanetwopen-e269753-s001.pdf]

Supplementary Online Content

Joshi S, Jing M, Wheeler-Martin K, et al. Driving time, distance, and cost to access syringe services programs in the US. *JAMA Netw Open*. 2026;9(4):e269753. doi:10.1001/jamanetworkopen.2026.9753

eFigure 1. Flowchart of SSP Addresses Included in the Final Analysis

eFigure 2. Flowchart of Population-Weighted Centroids of Census Tracts Included in the Final Analysis

eTable 1. Population-Weighted Driving Times and Distances to the Closest SSPs at the State Level Excluding Census Tracts With Routes That Take Longer Than 12 Hours

eTable 2. Population-Weighted Driving Costs to the Closest SSPs at the State Level Excluding Census Tracts With Routes That Take Longer Than 12 Hours

eTable 3. Driving Times and Driving Distances to the Closest SSPs Without Excluding Census Tracts With Routes That Take Longer Than 12 Hours

eTable 4. Driving Costs to the Closest SSPs Without Excluding Census Tracts With Routes That Take Longer Than 12 Hours

This supplementary material has been provided by the authors to give readers additional information about their work.

© 2026 Joshi S et al. *JAMA Network Open*.

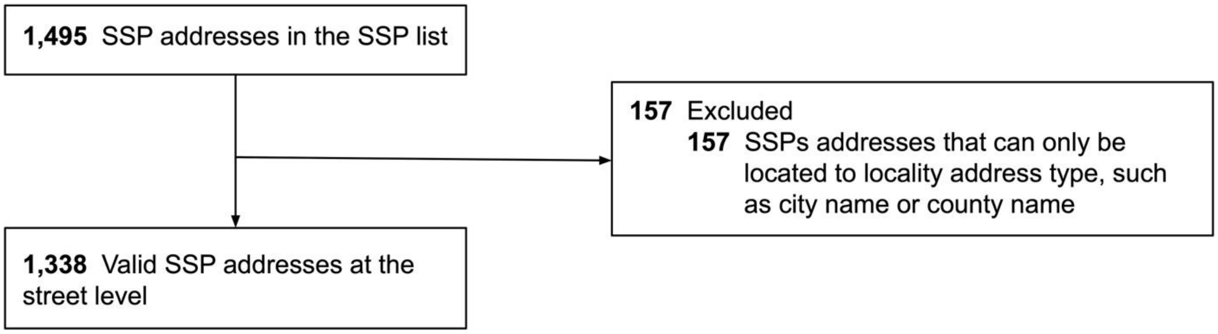

eFigure 1. Flowchart of SSP addresses included in the final analysis

© 2026 Joshi S et al. *JAMA Network Open*.

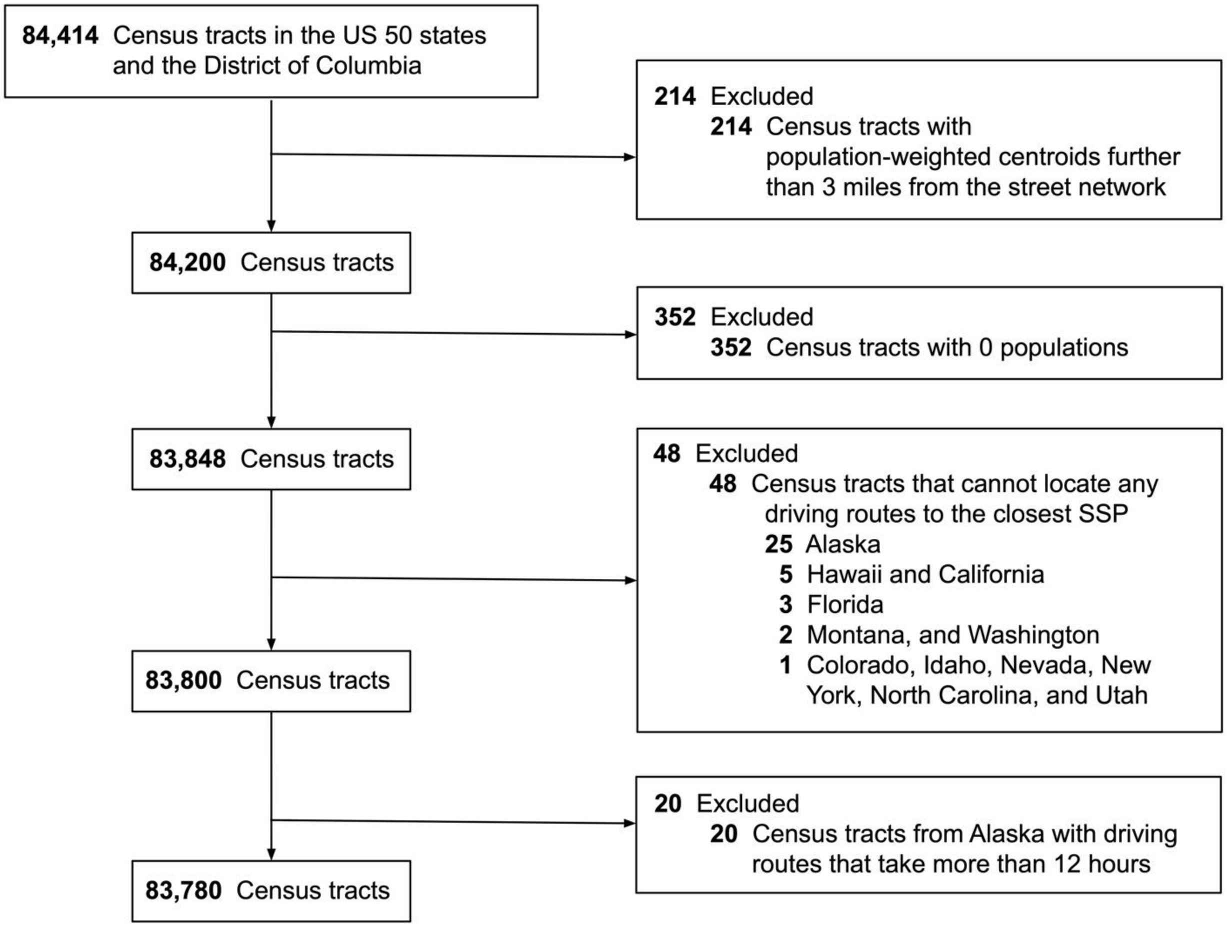

eFigure 2. Flowchart of population-weighted centroids of census tracts included in the final analysis

© 2026 Joshi S et al. *JAMA Network Open*.

eTable 1. Population-weighted driving times and distances to the closest SSPs at the state level excluding census tracts with routes that take longer than 12 hours

	One-way driving time, min		One-way driving distance, mile	
**State**	**Population-weighted mean (95% CI)**	**Median [IQR]**	**Population-weighted mean (95% CI)**	**Median [IQR]**
**At the census tract level (N = 83,780)**				
Total US	46.1 (45.7 - 46.5)	23.3 [12.2 - 58.5]	41.8 (41.3 - 42.2)	15.35 [5.5 - 50.5]
Alabama (N = 1,434)	142.7 (140.4, 144.9)	138.9 [114.2 - 164.9]	147.5 (145.0, 149.9)	145.5 [113.7 - 173.7]
Alaska (N = 132)	27.8 (19.4, 36.1)	12.2 [7.6 - 22.6]	17.2 (11.4, 23.0)	6.3 [3.0 - 14.4]
Arizona (N = 1,753)	29.2 (27.9, 30.5)	20.5 [13.1 - 34.1]	21.7 (20.5, 23.0)	13.1 [6.2 - 24.6]
Arkansas (N = 823)	83.0 (79.6, 86.5)	82.9 [44.5 - 115.3]	81.0 (77.6, 84.4)	83.8 [42.3 - 114.3]
California (N = 9,092)	18.8 (18.5, 19.1)	14.9 [9.2 - 22.8]	12.3 (12.0, 12.6)	7.9 [3.4 - 15.3]
Colorado (N = 1,438)	21.2 (20.2, 22.2)	16.0 [9.8 - 24.2]	14.5 (13.6, 15.5)	8.9 [4.0 - 16.7]
Connecticut (N = 876)	14.4 (13.9, 14.9)	13.8 [8.3 - 19.4]	8.1 (7.7, 8.5)	7.1 [2.8 - 12.1]
Delaware (N = 258)	19.1 (17.9, 20.4)	17.1 [12.2 - 26.8]	11.3 (10.3, 12.3)	8.7 [5.2 - 18.4]
District of Columbia (N = 206)	6.4 (5.8, 7.0)	5.3 [3.3 - 7.9]	1.4 (1.3, 1.6)	1.0 [0.6 - 1.7]
Florida (N = 5,091)	64.0 (62.2, 65.9)	40.8 [18.9 - 93.2]	59.2 (57.2, 61.3)	31.7 [9.6 - 83.6]
Georgia (N = 2,784)	55.2 (53.3, 57.2)	35.1 [20.6 - 85.0]	48.0 (45.9, 50.0)	24.4 [11.7 - 78.4]
Hawaii (N = 427)	21.1 (19.4, 22.8)	17.0 [10.9 - 26.3]	10.9 (9.9, 12.0)	8.3 [3.3 - 14.0]
Idaho (N = 455)	35.6 (32.3, 38.9)	19.2 [9.5 - 58.6]	33.0 (29.2, 36.8)	11.6 [4.0 - 53.7]
Illinois (N = 3,261)	23.7 (23.0, 24.3)	17.7 [9.0 - 32.7]	17.0 (16.3, 17.6)	9.2 [3.1 - 24.0]
Indiana (N = 1,689)	34.2 (33.2, 35.3)	29.9 [16.1 - 50.9]	26.2 (25.2, 27.2)	21.4 [8.5 - 41.7]
Iowa (N = 894)	76.9 (73.6, 80.3)	83.2 [46.5 - 109.7]	74.8 (71.3, 78.4)	82.2 [41.7 - 107.6]
Kansas (N = 825)	191.4 (188.4, 194.4)	191.4 [149.9 - 222.3]	213.0 (209.6, 216.4)	210.3 [170.4 - 245.8]

© 2026 Joshi S et al. *JAMA Network Open*.

State	One-way driving time, min		One-way driving distance, mile	
	Population-weighted mean (95% CI)	Median [IQR]	Population-weighted mean (95% CI)	Median [IQR]
Kentucky (N = 1,304)	16.8 (16.2, 17.4)	15.4 [8.1 - 23.1]	11.3 (10.7, 11.8)	9.3 [3.5 - 16.7]
Louisiana (N = 1,370)	47.8 (45.9, 49.8)	38.7 [13.9 - 73.1]	41.5 (39.6, 43.5)	31.5 [7.6 - 67.2]
Maine (N = 400)	31.7 (28.4, 35.0)	24.9 [11.9 - 40.7]	23.1 (19.8, 26.3)	16.2 [5.7 - 27.0]
Maryland (N = 1,460)	14.8 (14.3, 15.3)	12.3 [7.6 - 18.5]	8.1 (7.7, 8.5)	5.3 [2.7 - 10.5]
Massachusetts (N = 1,606)	17.5 (16.4, 18.6)	15.3 [8.9 - 22.0]	8.4 (8.0, 8.8)	6.2 [2.5 - 11.7]
Michigan (N = 2,933)	16.3 (15.9, 16.8)	12.2 [7.1 - 21.0]	11.7 (11.2, 12.1)	7.0 [3.1 - 15.7]
Minnesota (N = 1,501)	31.6 (30.2, 33.0)	21.6 [11.3 - 48.2]	26.6 (25.2, 28.1)	15.6 [5.7 - 43.4]
Mississippi (N = 874)	119.5 (115.9, 123.1)	118.9 [82.2 - 163.2]	124.2 (120.1, 128.3)	120.4 [82.0 - 176.0]
Missouri (N = 1,654)	108.8 (105.3, 112.2)	129.5 [37.4 - 172.6]	113.4 (109.3, 117.4)	132.4 [28.8 - 190.4]
Montana (N = 317)	100.2 (92.3, 108.1)	90.2 [57.3 - 169.6]	107.6 (98.1, 117.1)	86.9 [51.2 - 173.8]
Nebraska (N = 553)	172.9 (168.6, 177.2)	165.7 [130.1 - 211.3]	192.2 (187.1, 197.2)	184.9 [142.7 - 229.8]
Nevada (N = 771)	27.7 (25.4, 30.0)	19.4 [13.1 - 26.9]	22.0 (19.3, 24.6)	12.2 [7.2 - 19.7]
New Hampshire (N = 348)	19.4 (18.0, 20.7)	17.2 [9.5 - 27.6]	11.9 (10.8, 13.0)	9.4 [3.7 - 17.6]
New Jersey (N = 2,175)	22.7 (22.2, 23.2)	19.9 [13.1 - 29.5]	14.3 (13.8, 14.8)	10.9 [4.9 - 20.7]
New Mexico (N = 609)	14.0 (12.9, 15.1)	10.8 [5.7 - 18.4]	8.4 (7.5, 9.3)	5.2 [2.1 - 10.7]
New York (N = 5,357)	16.6 (16.2, 17.0)	12.9 [7.6 - 21.6]	9.2 (8.9, 9.6)	4.5 [1.7 - 13.2]
North Carolina (N = 2,654)	21.2 (20.7, 21.7)	19.3 [12.2 - 29.1]	14.2 (13.7, 14.6)	12.0 [5.9 - 20.8]
North Dakota (N = 228)	38.9 (33.2, 44.5)	36.4 [10.9 - 75.2]	35.2 (29.2, 41.3)	33.9 [5.1 - 77.4]
Ohio (N = 3,158)	21.7 (21.2, 22.2)	17.0 [10.2 - 28.0]	15.8 (15.4, 16.3)	10.7 [4.7 - 21.7]
Oklahoma (N = 1,204)	46.9 (44.6, 49.3)	30.2 [16.3 - 74.3]	45.3 (42.8, 47.9)	24.9 [10.8 - 77.9]
Oregon (N = 993)	27.1 (25.6, 28.6)	19.2 [9.0 - 38.6]	18.8 (17.4, 20.1)	10.5 [3.3 - 27.4]

© 2026 Joshi S et al. *JAMA Network Open*.

State	One-way driving time, min		One-way driving distance, mile	
	Population-weighted mean (95% CI)	Median [IQR]	Population-weighted mean (95% CI)	Median [IQR]
Pennsylvania (N = 3,431)	40.8 (39.8, 41.8)	36.1 [16.4 - 61.9]	31.2 (30.2, 32.2)	24.0 [6.7 - 52.3]
Rhode Island (N = 246)	18.9 (17.3, 20.5)	17.5 [9.4 - 25.9]	10.0 (8.9, 11.1)	7.8 [2.9 - 15.5]
South Carolina (N = 1,314)	75.0 (72.4, 77.6)	82.3 [37.3 - 113.5]	62.7 (60.3, 65.1)	62.4 [28.0 - 98.9]
South Dakota (N = 242)	202.5 (193.2, 211.8)	182.1 [152.1 - 267.2]	216.9 (206.5, 227.4)	195.8 [158.4 - 283.4]
Tennessee (N = 1,693)	27.2 (26.3, 28.1)	23.2 [12.1 - 38.5]	20.3 (19.4, 21.1)	15.7 [6.1 - 29.9]
Texas (N = 6,868)	123.4 (121.8, 125.0)	144.4 [91.6 - 163.0]	131.5 (129.7, 133.3)	154.5 [92.2 - 177.8]
Utah (N = 712)	34.0 (31.2, 36.8)	21.4 [11.8 - 40.1]	30.2 (27.1, 33.4)	14.6 [5.8 - 35.7]
Vermont (N = 192)	22.9 (20.7, 25.1)	23.0 [12.6 - 35.6]	15.0 (13.2, 16.7)	14.0 [6.4 - 24.6]
Virginia (N = 2,175)	47.6 (46.4, 48.7)	47.8 [27.0 - 64.1]	39.1 (37.9, 40.2)	38.2 [17.3 - 56.1]
Washington (N = 1,769)	19.5 (18.8, 20.2)	16.6 [10.0 - 24.2]	11.9 (11.4, 12.4)	9.4 [4.0 - 15.8]
West Virginia (N = 546)	26.1 (24.7, 27.5)	22.7 [12.7 - 38.0]	18.0 (16.8, 19.2)	15.0 [6.2 - 27.3]
Wisconsin (N = 1,526)	24.3 (23.5, 25.1)	20.1 [10.3 - 36.9]	18.7 (17.9, 19.5)	13.5 [4.5 - 30.0]
Wyoming (N = 160)	166.9 (152.9, 181.0)	162.6 [87.0 - 250.6]	183.1 (166.0, 200.1)	167.6 [79.1 - 251.5]

© 2026 Joshi S et al. *JAMA Network Open*.

eTable 2. Population-weighted driving costs to the closest SSPs at the state level excluding census tracts with routes that take longer than 12 hours

State	Estimation by 2024 IRS deduction for mileage (1-way driving cost), US dollar ($)		Estimation by 2022 state mean cost of gasoline (1-way fuel cost), US dollar ($)	
	**Population-weighted mean (95% CI)**	**Median [IQR]**	**Population-weighted mean (95% CI)**	**Median [IQR]**
**At the census tract level (N = 83,780)**				
Total US	8.77 (8.68 - 8.86)	3.22 [1.15 - 10.61]	6.91 (6.84 - 6.98)	2.76 [0.99 - 8.68]
Alabama (N = 1,434)	30.97 (30.46, 31.48)	30.56 [23.89 - 36.48]	23.06 (22.68, 23.43)	22.75 [17.78 - 27.16]
Alaska (N = 132)	3.61 (2.38, 4.83)	1.32 [0.63 - 3.02]	4.27 (2.82, 5.72)	1.57 [0.75 - 3.58]
Arizona (N = 1,753)	4.57 (4.30, 4.83)	2.74 [1.31 - 5.17]	4.35 (4.09, 4.60)	2.61 [1.24 - 4.92]
Arkansas (N = 823)	17.01 (16.29, 17.72)	17.60 [8.88 - 24.00]	12.81 (12.27, 13.35)	13.26 [6.69 - 18.08]
California (N = 9,092)	2.59 (2.53, 2.65)	1.65 [0.71 - 3.22]	2.84 (2.78, 2.91)	1.82 [0.78 - 3.54]
Colorado (N = 1,438)	3.05 (2.85, 3.25)	1.87 [0.85 - 3.50]	2.48 (2.32, 2.64)	1.52 [0.69 - 2.85]
Connecticut (N = 876)	1.71 (1.62, 1.79)	1.50 [0.58 - 2.55]	1.48 (1.41, 1.56)	1.30 [0.50 - 2.21]
Delaware (N = 258)	2.38 (2.17, 2.59)	1.83 [1.10 - 3.86]	2.00 (1.82, 2.17)	1.54 [0.93 - 3.24]
District of Columbia (N = 206)	0.30 (0.27, 0.34)	0.21 [0.13 - 0.36]	0.27 (0.24, 0.30)	0.19 [0.12 - 0.31]
Florida (N = 5,091)	12.44 (12.00, 12.87)	6.66 [2.02 - 17.55]	9.42 (9.09, 9.75)	5.05 [1.53 - 13.29]
Georgia (N = 2,784)	10.07 (9.63, 10.51)	5.12 [2.46 - 16.46]	7.32 (7.01, 7.64)	3.72 [1.79 - 11.97]
Hawaii (N = 427)	2.29 (2.07, 2.52)	1.73 [0.69 - 2.94]	2.72 (2.45, 2.98)	2.05 [0.82 - 3.49]
Idaho (N = 455)	6.93 (6.13, 7.73)	2.43 [0.85 - 11.29]	6.15 (5.44, 6.86)	2.16 [0.76 - 10.02]
Illinois (N = 3,261)	3.56 (3.42, 3.71)	1.93 [0.65 - 5.04]	2.89 (2.77, 3.01)	1.56 [0.53 - 4.09]
Indiana (N = 1,689)	5.51 (5.29, 5.72)	4.49 [1.78 - 8.76]	4.32 (4.15, 4.48)	3.52 [1.40 - 6.86]
Iowa (N = 894)	15.72 (14.97, 16.47)	17.25 [8.76 - 22.61]	12.53 (11.93, 13.12)	13.75 [6.99 - 18.02]
Kansas (N = 825)	44.73 (44.01, 45.45)	44.15 [35.79 - 51.61]	35.42 (34.85, 35.99)	34.96 [28.34 - 40.86]

© 2026 Joshi S et al. *JAMA Network Open*.

State	Estimation by 2024 IRS deduction for mileage (1-way driving cost), US dollar ($)		Estimation by 2022 state mean cost of gasoline (1-way fuel cost), US dollar ($)	
	Population-weighted mean (95% CI)	Median [IQR]	Population-weighted mean (95% CI)	Median [IQR]
Kentucky (N = 1,304)	2.36 (2.26, 2.47)	1.96 [0.73 - 3.52]	1.92 (1.83, 2.00)	1.59 [0.59 - 2.85]
Louisiana (N = 1,370)	8.72 (8.31, 9.13)	6.61 [1.59 - 14.12]	6.53 (6.22, 6.84)	4.94 [1.19 - 10.57]
Maine (N = 400)	4.85 (4.16, 5.53)	3.41 [1.19 - 5.67]	4.18 (3.59, 4.78)	2.94 [1.03 - 4.89]
Maryland (N = 1,460)	1.70 (1.62, 1.78)	1.11 [0.56 - 2.20]	1.44 (1.37, 1.50)	0.94 [0.47 - 1.86]
Massachusetts (N = 1,606)	1.76 (1.69, 1.84)	1.31 [0.52 - 2.46]	1.52 (1.45, 1.58)	1.13 [0.45 - 2.12]
Michigan (N = 2,933)	2.45 (2.36, 2.55)	1.48 [0.66 - 3.29]	1.94 (1.87, 2.02)	1.17 [0.52 - 2.61]
Minnesota (N = 1,501)	5.60 (5.29, 5.90)	3.28 [1.20 - 9.11]	4.54 (4.30, 4.79)	2.66 [0.98 - 7.40]
Mississippi (N = 874)	26.08 (25.22, 26.95)	25.28 [17.22 - 36.97]	19.49 (18.84, 20.13)	18.88 [12.87 - 27.62]
Missouri (N = 1,654)	23.81 (22.96, 24.66)	27.80 [6.04 - 39.98]	18.42 (17.76, 19.08)	21.50 [4.67 - 30.93]
Montana (N = 317)	22.60 (20.60, 24.60)	18.26 [10.75 - 36.49]	19.98 (18.21, 21.75)	16.14 [9.50 - 32.27]
Nebraska (N = 553)	40.36 (39.30, 41.42)	38.82 [29.97 - 48.27]	33.05 (32.18, 33.92)	31.79 [24.55 - 39.53]
Nevada (N = 771)	4.61 (4.05, 5.17)	2.57 [1.51 - 4.13]	4.57 (4.02, 5.13)	2.54 [1.50 - 4.10]
New Hampshire (N = 348)	2.51 (2.28, 2.74)	1.98 [0.77 - 3.69]	2.10 (1.91, 2.29)	1.66 [0.64 - 3.09]
New Jersey (N = 2,175)	3.00 (2.89, 3.10)	2.28 [1.02 - 4.36]	2.44 (2.35, 2.52)	1.85 [0.83 - 3.54]
New Mexico (N = 609)	1.76 (1.57, 1.95)	1.09 [0.44 - 2.25]	1.37 (1.22, 1.52)	0.85 [0.34 - 1.75]
New York (N = 5,357)	1.94 (1.87, 2.01)	0.95 [0.35 - 2.78]	1.57 (1.52, 1.63)	0.77 [0.28 - 2.25]
North Carolina (N = 2,654)	2.97 (2.89, 3.06)	2.52 [1.25 - 4.36]	2.33 (2.26, 2.40)	1.98 [0.98 - 3.42]
North Dakota (N = 228)	7.39 (6.12, 8.66)	7.12 [1.07 - 16.26]	6.26 (5.18, 7.33)	6.03 [0.90 - 13.76]
Ohio (N = 3,158)	3.33 (3.23, 3.42)	2.25 [1.00 - 4.55]	2.69 (2.61, 2.77)	1.82 [0.81 - 3.68]
Oklahoma (N = 1,204)	9.52 (8.98, 10.06)	5.22 [2.26 - 16.37]	7.38 (6.96, 7.80)	4.05 [1.75 - 12.69]

© 2026 Joshi S et al. *JAMA Network Open*.

State	Estimation by 2024 IRS deduction for mileage (1-way driving cost), US dollar ($)		Estimation by 2022 state mean cost of gasoline (1-way fuel cost), US dollar ($)	
	**Population-weighted mean (95% CI)**	**Median [IQR]**	**Population-weighted mean (95% CI)**	**Median [IQR]**
Oregon (N = 993)	3.95 (3.66, 4.23)	2.20 [0.68 - 5.76]	4.01 (3.72, 4.30)	2.24 [0.70 - 5.86]
Pennsylvania (N = 3,431)	6.55 (6.34, 6.75)	5.05 [1.40 - 10.98]	5.64 (5.47, 5.82)	4.35 [1.21 - 9.47]
Rhode Island (N = 246)	2.09 (1.86, 2.32)	1.65 [0.60 - 3.26]	1.80 (1.61, 2.00)	1.42 [0.52 - 2.81]
South Carolina (N = 1,314)	13.16 (12.66, 13.66)	13.10 [5.88 - 20.78]	9.71 (9.34, 10.08)	9.66 [4.33 - 15.32]
South Dakota (N = 242)	45.56 (43.36, 47.75)	41.12 [33.26 - 59.51]	37.58 (35.77, 39.39)	33.92 [27.43 - 49.08]
Tennessee (N = 1,693)	4.26 (4.08, 4.44)	3.29 [1.27 - 6.28]	3.35 (3.21, 3.49)	2.59 [1.00 - 4.94]
Texas (N = 6,868)	27.62 (27.24, 27.99)	32.45 [19.35 - 37.33]	20.39 (20.11, 20.67)	23.96 [14.29 - 27.56]
Utah (N = 712)	6.35 (5.68, 7.02)	3.06 [1.22 - 7.51]	5.61 (5.02, 6.20)	2.71 [1.08 - 6.63]
Vermont (N = 192)	3.14 (2.77, 3.51)	2.94 [1.35 - 5.16]	2.73 (2.40, 3.05)	2.55 [1.17 - 4.48]
Virginia (N = 2,175)	8.20 (7.96, 8.44)	8.03 [3.63 - 11.79]	6.24 (6.06, 6.42)	6.11 [2.76 - 8.97]
Washington (N = 1,769)	2.50 (2.39, 2.61)	1.97 [0.85 - 3.31]	2.43 (2.32, 2.54)	1.91 [0.82 - 3.22]
West Virginia (N = 546)	3.77 (3.52, 4.02)	3.16 [1.29 - 5.73]	3.10 (2.89, 3.31)	2.59 [1.06 - 4.71]
Wisconsin (N = 1,526)	3.92 (3.75, 4.10)	2.84 [0.95 - 6.29]	3.29 (3.14, 3.43)	2.38 [0.80 - 5.27]
Wyoming (N = 160)	38.45 (34.86, 42.03)	35.19 [16.61 - 52.81]	31.82 (28.86, 34.79)	29.12 [13.75 - 43.71]

© 2026 Joshi S et al. *JAMA Network Open*.

eTable 3. Driving times and driving distances to the closest SSPs without excluding census tracts with routes that take longer than 12 hours

Outcome	One-way driving time, min		One-way driving distance, mile	
	**Population-weighted mean (95% CI)**	**Median [IQR]**	**Population-weighted mean (95% CI)**	**Median [IQR]**
**At the census tract level (N = 83,800[1])**				
Total US	46.6 (46.0 - 47.2)	23.3 [12.2 - 58.6]	41.8 (41.4 - 42.3)	15.4 [5.5 - 50.6]
**NCHS Urban-Rural County Classification (N = 83,800[1])**				
Metropolitan counties (N = 70,008)				
Large central metro (N = 25,529)	31.8 (31.1 - 32.4)	14.0 [8.2 - 22.2]	27.5 (26.8 - 28.3)	6.5 [2.5 - 14.2]
Large fringe metro (N = 19,256)	38.0 (37.4 - 38.7)	24.6 [16.0 - 38.7]	31.7 (31.0 - 32.5)	16.1 [8.1 - 29.5]
Medium metro (N =17,666)	51.7 (50.9 - 52.6)	26.8 [12.6 - 79.4]	48.3 (47.3 - 49.3)	18.6 [6.4 - 75.1]
Small metro (N = 7,557)	60.2 (58.7 - 61.7)	41.3 [14.1 - 89.2]	57.5 (55.8 - 59.1)	33.8 [7.9 - 86.2]
Nonmetropolitan counties (N = 13,792[1])				
Micropolitan (N = 7,905[2])	69.8 (68.0 - 71.6)	51.4 [25.1 - 96.4]	65.2 (63.6 - 66.9)	44.1 [17.8 - 91.1]
Noncore (N = 5,887[3])	94.8 (86.5 - 103.1)	71.9 [41.1 - 128.0]	84.9 (82.9 - 87.0)	63.7 [32.3 - 126.0]
**SSPs legal (N = 83,800[1])**				
SSP permitted (N = 66,865[1])	30.7 (30.1 - 31.3)	19.1 [10.7 - 36.1]	23.7 (23.4 - 23.9)	11.5 [4.4 - 27.4]
SSP prohibited (N = 16,935)	110.7 (109.6 -111.8)	122.8 [49.8 - 161.5]	115.2 (113.9 -116.5)	127.8 [40.3 - 175.4]

[1] An addition of 20 Alaska census tracts with driving routes exceeding 12 hours.

[2] An addition of 5 Alaska census tracts with driving routes exceeding 12 hours.

[3] An addition of 15 Alaska census tracts with driving routes exceeding 12 hours.

© 2026 Joshi S et al. *JAMA Network Open*.

eTable 4. Driving costs to the closest SSPs without excluding census tracts with routes that take longer than 12 hours

Outcome	Estimation by 2024 IRS deduction for mileage (1-way driving cost), US dollar ($)		Estimation by 2022 state mean cost of gasoline (1-way fuel cost), US dollar ($)	
	population-weighted mean (95% CI)	median [IQR]	population-weighted mean (95% CI)	median [IQR]
**At the census tract level (N = 83,800[1])**				
Total US	8.79 (8.70 - 8.89)	3.22 [1.15 - 10.62]	6.93 (6.86 – 7.00)	2.76 [0.99 - 8.69]
**NCHS Urban-Rural County Classification (N = 83,800[1])**				
Metropolitan counties (N = 70,008)				
Large central metro (N = 25,529)	5.78 (5.63 - 5.93)	1.37 [0.53 - 2.99]	4.55 (4.44 - 4.66)	1.22 [0.47 - 2.72]
Large fringe metro (N = 19,256)	6.67 (6.52 - 6.82)	3.38 [1.70 - 6.20]	5.25 (5.13 - 5.36)	2.80 [1.41 - 4.99]
Medium metro (N =17,666)	10.15 (9.94 - 10.35)	3.91 [1.34 - 15.77]	7.93 (7.78 - 8.09)	3.37 [1.14 - 12.47]
Small metro (N = 7,557)	12.07 (11.72 - 12.41)	7.11 [1.66 - 18.11]	9.60 (9.33 - 9.88)	5.94 [1.39 - 14.15]
Nonmetropolitan counties (N = 13,792[1])				
Micropolitan (N = 7,905[2])	13.70 (13.36 - 14.04)	9.25 [3.75 - 19.12]	10.93 (10.65 - 11.22)	7.47 [3.11 - 15.33]
Noncore (N = 5,887[3])	17.83 (17.40 - 18.26)	13.38 [6.78 - 26.47]	14.18 (13.81 - 14.54)	10.57 [5.55 - 20.72]
**SSPs legal (N = 83,780[1])**				
SSP permitted (N = 66,865[1])	4.97 (4.91 - 5.03)	2.41 [0.93 - 5.74]	4.08 (4.03 – 4.13)	2.08 [0.80 - 4.81]
SSP prohibited (N = 16,935)	24.19 (23.92 - 24.46)	26.84 [8.46 - 36.82]	17.82 (17.63 - 18.02)	20.27 [6.97 - 27.56]

[1] An addition of 20 Alaska census tracts with driving routes exceeding 12 hours.

[2] An addition of 5 Alaska census tracts with driving routes exceeding 12 hours.

[3] An addition of 15 Alaska census tracts with driving routes exceeding 12 hours.

© 2026 Joshi S et al. *JAMA Network Open*.
