# Supplementary material for: Driving Time, Distance, and Cost to Access Syringe Services Programs in the US
Source: JAMA Netw Open. 2026 Apr 29;9(4):e269753. doi: 10.1001/jamanetworkopen.2026.9753 (PMC13129881; doi:10.1001/jamanetworkopen.2026.9753)
Supplement: Supplement 2. — Data Sharing Statement [file jamanetwopen-e269753-s002.pdf]

Data Sharing Statement

Joshi. Driving Time, Distance, and Cost to Access Syringe Services Programs in the US. *JAMA Netw Open*. Published April 29, 2026. doi:10.1001/jamanetworkopen.2026.9753

Data
Data available: No
